# Recognising forced migrants in transnational social work

**DOI:** 10.1108/IJMHSC-11-2016-0042

**Published:** 2018-06-11

**Authors:** Kati Turtiainen

**Affiliations:** Department of Social Sciences, University of Jyväskylä, Kokkola University Consortium Chydenius, Kokkola, Finland

**Keywords:** Social work, Forced migration, Asylum seekers, Recognition, Transmigration, Undocumented migrants

## Abstract

**Purpose:**

Nation states’ neoliberal policies do not regard asylum seekers and undocumented migrants as deserving of a good life. Social work in welfare states is highly connected to the policies of nation states. There is a need to address theories in social work that have a transnational focus at the local level. Axel Honneth’s recognition theory enables an approach to forced migration from the direction of personal relations and personhood itself. The core idea is that if people cannot gain recognition, this causes harm to their self-realisation. The purpose of this paper is discuss how the recognition theory overcomes a national focus in social work.

**Design/methodology/approach:**

This paper is theoretical. The relations of recognition are discussed in the context of transnational social work in welfare states with forced migrants.

**Findings:**

The theory of recognition in social work practice with people who do not have a residence permit is best articulated by an understanding of rights concerning all the attributes of the person, i.e. as a needy being, autonomous and particular in a community.

**Originality/value:**

Forced migrants’ backgrounds provide a specific backdrop for misrecognition, which may harm self-relations. The relations of recognition contribute to social work by providing the sensitivity required to evaluate the complexity of views and attitudes that affect the way we encounter service users. The relations of recognition (care, respect and esteem) give normative criteria for communication in order to take another person as a person, which, in turn, contributes to healthy self-relations of forced migrants.

## Introduction

The current features of forced migration are in flux due to different human rights abuses worldwide. There is a close link between processes of globalisation, underdevelopment, impoverishment, poor governance, endemic conflict and the human rights’ abuses that force people to leave their habitual residences (Castles and Miller, 2009). By the end of 2016, a total of 65.6 million individuals worldwide were forcibly displaced, although, not all forced migrants were crossing international state borders. Many were forced to move within their own countries in order to avoid the effects of armed conflict, situations of generalised violence, or natural and manmade disasters, and of this, total 43 million were internally displaced people (IDPs). At the same time, 2.8 million people decided to seek asylum in Europe. The countries hosting people in need of protection are often the poorest countries in the world (UNHCR, 2016). With no legal opportunities or future prospects available, many displaced people follow treacherous sea routes, and encounter exploitation and life-threatening dangers at the hands of smuggling networks. In this paper, the main focus is the situation of asylum seekers in Europe, as well as those whose asylum application has been rejected. They will be referred to as undocumented migrants.

The protection needs of forced migrants, such as refugees, asylum seekers and IDPs, presents challenges for the international community and nation states. The European Union, for example, tends to prevent people from migrating by using active containment policies and practices, which worsen civil insecurity in many regions, particularly the Middle East and North Africa (Held, 2016). While the EU is blocking mobility to the north, the so-called “migration industry” is increasing as people risk more dangerous routes and are dependent on powerful smuggling networks (Andersson, 2016). At the same time, more than 5,000 lives have been lost in the Mediterranean (IOM, 2017). Once asylum seekers reach the EU, they may face totally inhuman treatment, as is the case in the Balkans (Zaviršek, 2017), or they may manage to reach Italy or Greece where they are left waiting for relocation to other European states.

About 40 per cent of asylum applications were rejected as a first decision in EU28 countries, affecting more than 400,000 people altogether (Eurostat, 2017). After the initial rejection of an asylum application, many asylum seekers find it better to try and survive in Europe without protection from a nation state than to go back to the life-threatening conditions they have escaped from. They are forced to maintain their lives in transnational conditions, as undocumented persons, without having residency anywhere. They are also extremely vulnerable to blackmail and exploitation by employers. Transnational life may become a strategy of survival, as, at the same time, they have to maintain emotional, family, financial, moral ties in many countries (Hunter *et al.*, 2010; Faist *et al.*, 2013). They may lack a long-term residential base, which means that they are continuously living in different countries and cultures (Furman *et al.*, 2010). In general, transmigrants – voluntary or forced – are involved with two or more distinct national environments. They engage in processes of transnationalism in the economic, cultural and social fields that cross national borders (Levitt and Glick Schiller, 2004; Vertovec, 2009).

In the EU, the policies and practises of many nation states do not show solidarity with asylum seekers or respect their human rights, but instead demonstrate xenophobic attitudes towards them. The sovereignty of the nation state seems to be more important than extending mutuality to unprotected people (Benhabib, 2004). This is a consequence of the fragmentation of values and solidarity between EU member states, leading to an ideology of competitiveness and productivity by which forced migrants are considered as a burden (Blomberg *et al.*, 2013). Thus, forced migrants, and especially undocumented people, illustrate the human rights violations in the world today and the lack of protection offered by the international community (Turton, 2003). Therefore, social work with asylum seekers and undocumented migrants has to transcend the dominant national focus in order to overcome ethnocentric views, which are structured into the national policy framework within which social work delivers services (Wallimann, 2014). Furthermore, social work with a transnational population needs to address complex global and transnational problems (Furman *et al.*, 2010; Hugman *et al.*, 2010).

Thus, not only should social work practises transcend the national focus, but social work theories must also test their own knowledge base in order to avoid methodological nationalism. Methodological nationalism means that “state and society are held to be coterminous and territorially identical” (Faist *et al.*, 2013). In this theoretical paper, it is articulated that nation states’ borders are blocking individuals’ possibilities of full self-realisation and identity formation. Therefore, I apply Axel Honneth’s (1995) theory of the relations of recognition. His main idea is that the relations of care, respecting rights and social esteem, are a precondition for self-realisation. Analogously, disrespect is violating the development of positive self-relations and identity formation. Besides, by caring about the well-being of people, respecting their rights and esteeming their accomplishments, people are regarded as persons and not as mere instruments for other purposes. Refugees, asylum seekers and undocumented migrants have experiences of disrespect in their past, during their journeys to Europe and also in their new circumstances. The agency of undocumented people could be so limited that they may not have the facility to claim any rights and therefore they need people, such as social workers within social movements, to act on their behalf. These actions follow Honneth’s (2007) idea that the feeling of disrespect motivates the struggle for recognition, and, by doing so, adheres to the moral grammar of a good society.

Moreover, Honneth’s recognition theory is useful because “he rejects the liberal conception of human subjects as independent and self-determining” (Rossiter, 2014) and instead suggests that reciprocal relations of care, respect and social esteem renders people susceptible to mutual recognition. This notion is vital in societies where human beings are considered as consumers and subordinate to profits. In these circumstances, asylum seekers and undocumented migrants are “unwanted” and considered undeserving of (social) protection, and, as a result, they are cast out from the order of nation states.

I begin by describing migration and nation states in the global context. Next, I will address the principles of social work with a transnational population, especially with asylum seekers and undocumented migrants. Lastly, I will consider the relations of recognition applied to social work with asylum seekers and undocumented migrants in the nation state context, with reference to a number of cases from Finland. Finland is an example of “old” welfare state, which has subsequently shifted towards the violation of the human rights of asylum seekers.

## Globalisation, nation states and migration

Globalisation can be understood as the growing interdependence in economic, social, cultural, political and legal structures and global institutions. On one hand, globalisation is a rapid increase of cross-border flows of finance and trade, ideas, ideologies and knowledge about democratic and economic governance, cultural and media products, and people (Castles and Miller, 2009). On the other hand, globalisation is “a wild process involving interconnectedness and exclusion, integration and fragmentation, homogenization and diversity” (Kaldor, 2008, p. 271). For example, the globalisation of migration means more opportunities for some whereas others are forced to leave their habitual residences. The key factors of growing forced migration are “increasing international and domestic inequalities, the persistent demand for high- and low-skilled migrant labour in the segmented labour markets of wealthy societies, the lack of opportunities, population growth, oppression, and violent conflict in developing countries” (Held, 2016). Global and local inequalities are intertwined and affected by global power structures, which are highly connected to a neoliberal global economy and its policies. An important landmark in the accelerated expansion of neoliberal policies was the foundation of World Trade Organisation (WTO) after the fall of the Soviet regime in 1989. Besides the global economy, the WTO started to colonise the politics, education, culture, health, and also social welfare and social policy of nations (Meyer, 2009). However, current neoliberal policies are encountering obstacles because the globalisation of environmental degradation has been accelerated by resource-intensive growth in the developed world and because of the rise in global migration (Held, 2016). The main tension is sited between the maximisation of profits, the self-determination of nation states and servicing the needs of individuals as consumers (Harvey, 2011) rather than addressing the well-being of populations and sustainable development.

Within the struggles around self-determination, nation states have ceded their power to worldwide financial structures and processes, and they have also lost contact with the reality of migrants’ lives, which consist of multiple loyalties and identities in transnational spaces (Beck, 2006). As Sassen (2006, 2007) states, the dynamics of globalisation have radically also changed nation states. She uses the concept of denationalisation (2007), which captures processes that are localised in national settings, but that arise from the interaction of the global and the national. Moreover, Beck and Cronin (2012) write that the world system follows the logic of “global domestic politics”, where geographical, cultural, social and political separation between native and foreign is disintegrating. They describe how all actors, such as companies, religious communities, human rights’ movements and workers, as well as nationalists and terrorists are broadening their horizons of perception and action, adopting the perspectives of others and coordinating them for their own purposes. Paradoxically, nationalism has also been shattered in pieces by the globalisation of national. In practise, this has caused the disintegration and demise of globalism, which leads to the appearance of boundaries and borders, nationalism and protectionism, localism and ethnicity in the policies of nation states (Held and McCrew, 2007).

Migration is also affected by, and thus must be situated in, economic and political power structures. It has always been socially produced, patterned and embedded in specific historical phases (Sassen, 2006). Therefore, decisions by individual migrants, including forced migrants, must be situated in large constellations within broader dynamics. Furthermore, there has been an emergence of “new global classes” and one in particular that consists of disadvantaged low-wage workers, including members of transnational immigrant communities and households. Another is the transnational network of government officials – the so-called global elites – and yet another is a class of activists, including key sectors of global civil society and particular kinds of diasporic networks (Sassen, 2007).

During the last few years, migration patterns have changed due to increased forced migration. There is a nexus between failed international interventions, climate-induced displacement, civil conflicts, regional collapse and other widespread threats to life chances in many areas, particularly the Middle East and North Africa (e.g. Held, 2016). The term “mixed migration” or migration-asylum-nexus (e.g. Van Hear, 2011) is often used to refer to groups of people who are fleeing and seeking refuge or improvements in their lives. People from different backgrounds turn to smugglers, and so tread the same routes, and end up in the same countries and communities. For example, an enormous challenge arises from climate-induced displacement because millions of human beings will be displaced during the coming decades (IPCC, 1990). Kelley *et al.* (2015) state that manmade global warming will have an impact on future conflicts. For example, drought in Syria contributed to the causes of war there. Northern Syria’s agriculture was destroyed by the drought, which forced farmers to move to the cities, where poverty, government mismanagement and other factors created unrest that exploded in spring 2011 and caused millions of people to flee the country.

The Convention Relating to the Status of Refugees (UNHCR, 1951/1967) is a quite narrow legal category, and it does not meet the current protection needs of displaced people. This Convention was designed to deal with the large number of displaced people in Europe after the Second World War. The main idea was to give protection to those people who were persecuted by authoritarian states, but nowadays people are asking for protection more from fragile states (Betts, 2013). This means that people are fleeing because of human rights’ deprivations that are a result of the fact that weak states are unable or unwilling to ensure fundamental rights. IDPs, refugees recognised by UNHCR or UNRWA, and asylum seekers are different sides of the same coin, failed by inadequate regional and international durable solutions. There is even a lack of terminology to identify people who should have the entitlement not to be returned to their country of origin on human rights’ grounds (Betts, 2013). Betts suggests that these people might be referred to as “survival migrants” because the difference in rights and entitlements available to refugees compared with survival migrants fleeing serious deprivations is arbitrary. Betts proposes to change the perspective from the particular cause of movement to identifying a threshold of fundamental rights. If a person cannot survive without leaving a country, then distinguishing between persecution and other causes is meaningless from the individual’s perspective.

Responses to the needs of forced migrants are extremely scarce. In the EU, xenophobic attitudes and growing politicisation of migration and refugee issues have risen due to economic uncertainty. As Lorenz (2017) states, immigrants are “the symbolic carriers of that division” when inclusive welfare provisions have been reduced and the nations of the EU lack comprehensive social policies at a transnational level. Global neoliberal policies have been resisted by many countries, such as Finland, because comprehensive public welfare services were considered as an investment. However, during the past few decades, the attitude to European public welfare measures shifted towards being considered basically as a “burden”, and they experienced the effects of privatisation with an increasing dominance of ideas of competitiveness and productivity (Blomberg *et al.*, 2013). In addition, the solidarity between EU states has been fragmented, and there is no solidarity towards people seeking protection. For example, as a result of social and economic discrepancies, the EU is in a state of crisis. The current rise of right-wing movements throughout the EU is directly related to the erosion of the securities of the welfare state and the fragmentation of a shared understanding of welfare and social values (Lorenz, 2017). Nation states’ policies and their stakeholders are concern with competition for jobs and social services, and use the threat of international terrorism to raise anxiety about national security. One manifestation of the fragmentation of solidarity in the EU is the practical failure of a relocation programme for asylum seekers in Italy and Greece. In the EU, it was decided in 2015 that 160,000 asylum seekers from Italy and Greece would be relocated to different EU countries, but it is estimated that only about 25,000 were relocated up to the end of September 2017 (Eurostat, 2017). The commitment of many EU states to provide asylum has weakened since 2015 when the number of asylum seekers greatly increased. For example, some countries do not follow the non-refoulement principle under the European Convention on Human Rights and the UN Convention against Torture (de Weck, 2016). Accordingly, asylum seekers have fewer possibilities to use regular channels to seek asylum because nation states close their borders, use brutal containment measures and weaken laws for granting asylum.

## Social work practice with forced migrants in the transnational context

Historically, social work has a double heritage with regard to helping migrants in Europe. Waaldijk (2011) states that, on the one hand, social work had a background in Christian charity and helping the poor to become full citizens in democratic anti-fascist and anti-communist states, and, on the other hand, it also worked within the “systems that did not regard outsiders as citizens, but looked at them as colonial subjects, as inferior races, as class enemies or ‘unworthy’”. The individualising approaches of charity organisation were also “avoiding a fundamental debate on preventive social and labour policies” (Lorenz, 2017). Even if social work has such histories of abusive and racist aspects, social work pioneers were aware of transnational processes at the time (Lorenz, 2017). The history of social work practice as a modern profession is shaped and developed around the growth of welfare states (Esping-Andersen, 1990), which enabled the evolution of social work as a modern profession. With the increased professionalisation of social work and the emergence of the welfare state, the transnational perspective of social work lost its ground (Wallimann, 2014). The articulation of ethnic diversity in European societies, which occurred during the 1980s and 1990s, raised fundamental questions about the role of social work in society (Lorenz, 2006). Welfare policies had discriminatory effects, which reinforced the construction of immigrants as basically “undeserving” (Bommes and Geddes, 2000). Thus, nation state policies can come up in social work encounters with migrants, because state borders can “move” the everyday practices where the nation state project is discussed (Rigo, 2009; Balibar, 2004). As a result, social work practice can become nationalistic if it uses exclusive practices and “the unquestioned focus on the national dimension could even be labelled as ‘chauvinist social work’ given a trend towards transnational processes like migration” (Wallimann, 2014, p. 19).

This is an essential reminder for social work practice with asylum seekers, who are cast out of the current order of nation states. Social workers may prioritise the safeguarding of the nation state instead serving migrants (Lorenz, 2006, p. 67). Thus, social work with forced migrants tests the relationship of social work and the nation state. For example, Jönsson (2014) shows that in Swedish social work, the methods deployed with undocumented migrants are passive, legalistic and bureaucratic in their response. However, there are also helpful methods which improve social conditions for migrants by ignoring legal limitations. Furthermore, in Finland, social work practitioners may take part in processes of “othering” and using the illusion of equality, which can exclude those who do not fit into national ideals (e.g. Keskinen *et al.*, 2012; Keskinen, 2014; Anis, 2008).

In contrast, if we look at the global definitions of social work, migration is “a core business of social work” (Cox and Geisen, 2014, p. 161) because social workers are engaged with people in their localities and wider networks from nations to transnational spaces. The definition of social work (IASSW and IFSW, 2014) emphases that “[…] Principles of social justice, human rights, collective responsibility and respect for diversities are central to social work […]”. So, the definition gives the social worker a mandate as they are operating in a profession of human rights, which means they have a mandate beyond nation states. According to Staub-Bernasconi’s (e.g. 2014; also Ife, 2008; Reichert, 2011) comprehensive understanding of a human rights approach, the national law systems and their implementation is legitimated only if they do not contradict the supranational law of human rights. The mandate of human rights gives social workers a moral and ethical yardstick besides and beyond the state mandate. These mandates may contradict each other, and, as a result, the rights and needs of the transnational population may remain unmet because of laws and state policies. In addition, the relationship of global economic powers, which has changed the understanding of welfare states – especially the provision of social rights – “doesn’t care about the breakdown or destruction of social relations and social rules which guarantee needs-fulfilment for all individuals” (Staub-Bernasconi, 2014, p. 33). Asylum seekers and undocumented migrants increasingly lack all rights in Europe, which also has an enormous impact on their social relations and future prospects. In the next section, I will apply Honneth’s (1995, 2007) recognition theory to the notion of how the entirety of personhood and identity formation is threatened as a result of denying rights and other entitlements.

## Recognition as a source of the “social” and justice

Axel Honneth’s theory of recognition (also e.g. Taylor and Gutmann, 1994; Ikäheimo, 2003, 2008; Laitinen, 2003, 2009; McBride, 2009, 2013; Thompson, 2013; Seglow, 2009, 2010, 2016) focusses on a normative criteria of a good society, where normative issues and interpersonal relations are interrelated. Here, the core idea is that persons are justified in expecting respect, esteem and love or care from each other in order to maintain healthy self-respect, self-esteem and self-confidence. These self-attitudes contribute to a person’s ability for self-realisation. If a person cannot get recognition in a mutual relationship with others, their ability to govern themselves may be harmed (e.g. Ikäheimo, 2003). Moreover, if recognition is threatened and if these experiences are shared with other people, there is a potential for collective action and struggles to gain recognition. This notion is important in current societies, where people are considered as consumers as a result of neoliberal policies instead of consideration of their well-being as persons.

Burns and Thompson (2013) ask if the principles of recognition could be applied at the global level. Thompson (2013) argues that not all the elements of recognition theory can be scaled up to the global level. Therefore, Thompson combines Honneth’s recognition theory, which is a so-called identity model, together with Nancy Fraser’s “status model” of recognition, which does not refer to individual identity or self-realisation. Fraser focusses on a frame setting for whom and for how justice could be possible by dismantling institutional obstacles in order to enable full partners in social interaction. She calls her theory a post-Westphalian democratic justice (Fraser, 2007). In this, she focusses on the dimension of justice, which is concerned mainly with representation and is also a matter of social belonging (Fraser, 2007). Her three dimensional theory of justice includes, first, the cultural dimension of justice, such as racism, which corresponds to society’s “status order” and which can act as an obstacle to participatory parity. Second, the economic dimension corresponds to the “economic structure” of society. Third, the political dimension of justice corresponds to the “political constitution” of society, where just representation requires that the way in which political decisions are made and political boundaries are defined should enable participatory parity to be achieved (Fraser, 2007).

Many scholars claim that the recognition theory is useful to apply to social work. Rossiter (2014) states that the recognition theory “allows social work to situate its values in a direct and practical relationship to social justice” and it “rejects the liberal conception of human subjects as independent and self-determining, arguing that the inevitable dependence on others for identity formation renders people vulnerable to recognition”. Niemi (2014) writes that Honneth’s theory can help to formulate a conception of the “social” in social work. The “social” cannot remain bound by the goodwill of the people, but it plays a more fundamental role and has normative claims (Niemi, 2014, pp. 537-8). Accordingly, “the ideal of recognition is grounded in socially poor people’s feelings of disrespect” (Juul, 2009, p. 405). Moreover, Houston (2009, p. 1,288) has applied the recognition theory to social work by claiming that it can provide “a prism through which social workers can “tune in” to ethical imperatives”. Also, the relations of recognition have been considered a prerequisite and a tool for trust formation between resettled refugees and public authorities in Finland (Turtiainen, 2012).

In the next section, I discuss social work with forced migrants, especially undocumented migrants within the theoretical frame of Honneth’s relations of recognition, which are respect, care and social esteem. In addition, Fraser’s theory is applied, particularly in terms of frame setting.

### Respect

According to the theory of recognition, people must be respected, first, as having rights and entitlements that are juridically institutionalised, and, second, as being autonomous in everyday interactions. People need rights to secure their agency, and they need other people to confirm that they are competent agents and capable of making justifications (Honneth, 1995). For undocumented migrants, there are often no possibilities for claiming institutionalised juridical rights, which is “a political death” in Arendt’s (1968) words. A denial of rights is an example of what Fraser (2007) calls misframing, which leads people to suffer from this if the sovereignty of a nation-state denies them the access to enjoy human rights. In this sense, the EU “possesses a provincial soul” (Beck, 2005, p. 231).

Rights also give a moral basis for contributing to healthy self-respect. Seglow (2016) separates first, “agency self-respect”, which means that “through authoring their lives, individuals come to appreciate the value of their own agency, which they in turn have reason to respect”. A second dimension of self-respect is “entitlement self-respect”, which is entitled by the recognition of a person’s human rights, which should be institutionalised in the state level. With both dimensions of self-realisation, asylum seekers and undocumented migrants are vulnerable in front of national institutions and public authorities. Most obviously, respect, as a juridically institutionalised right, contradicts the most in the context of national social work with asylum seekers and undocumented migrants, who stay in the country without a residence permit. There is a contradiction also in international law as, on one hand, no international organisation has the formal responsibility for protecting people who have the human rights-based entitlement to not be returned home if they fall outside of the Convention Refugee Definition (Betts, 2013) Whilst, on the other hand, the Universal Declaration of Human Rights (1948) gives anyone the right to exit from their own country and the right to seek asylum from persecution. However, many countries weakened their laws for granting asylum after 2015 when larger numbers of asylum seekers come to Europe. For example, in Finland, the residence permit on humanitarian grounds ended.

The idea of global citizenship could be a direction to guarantee rights for millions of people who are waiting for state protection. If legal citizenship cannot be a source for claiming one’s rights to receive social care, then all human beings have the “right to have rights” on the basis of their common humanity and not their nationality (Moosa-Mitha, 2014; Isin *et al.*, 2008). The question of citizenship has been discussed in the framework of dual or multiple citizenships. Furthermore, many social work scholars *(e.g.* Ife, 2008; Staub-Bernasconi, 2014; Dominelli, 2010) state that global citizenship for forced migrants would be a considerable improvement of their rights. First of all, social work practice should receive a mandate from forced migrants and, if possible, in combination with (global) civil society (Staub-Bernasconi, 2014) and consider “asylum seeking as a political issue requiring direct political action” (Briskman, 2014, p. 308).

In the supranational level, there is a plan by the UN General Assembly to improve governance on migration. The so-called global compact for migration is planned to be the first, inter-governmentally negotiated agreement to cover all dimensions of international migration in a holistic and comprehensive manner, in order “to address the challenges associated with today’s migration, and to strengthen the contribution of migrants and migration to sustainable development” (UNHCR, 2017). The global compact is urgently needed in order to find a durable solution to the situation of those who do not meet the Refugee Convention 1951 requirements for being granted protection, who have been waiting for a solution in refugee camps and those who are of victims of development- and climate-induced displacement. The aim of the global compact is to include a broad coalition of actors and to address the needs of refugees and host communities. The aim is very contradictory to the notion of nation states’ reluctance to give protection and residencies to asylum seekers, which is addressed above.

### Care

Care is concerned with human beings for their own sake, regardless of who or what kind of person they are (Ikäheimo, 2003; Honneth, 1995). However, the recognition of the needs of forced migrants is often considered as instrumental in some other goal, usually economic, without regard for them as persons deserving of protection and a good life. In social work practice, caring for the well-being of people, and also taking care of people in personal and concrete ways, has been essential since the introduction of its pioneering practices (Niemi, 2014). Here, care as a recognitive attitude between social workers and their migrant service users is addressed from three perspectives. First, social workers must fight for the right to care for undocumented migrants without a residence permit. This practice incorporates the relation of respect and also Thompson’s (2013) understanding of care as a form of global justice, taking care of the needs of all people. Second, social workers must find and form personal relationships with forced migrants in order to show care by empathy, understanding and concrete actions. Third, social workers should find and fight for the possibilities of family reunification in order to enable private care relations. This practice also overlaps with the dimension of rights.

Expanding on the first of these, in order to take seriously the needs of undocumented migrants who do not have a residence permit, care must first of all be considered as the right to have access to social and health care. Thompson (2013) states that, if the relation of care is scaled from the local to the global level (here, beyond citizens of the state), care has to be transformed into the principle of needs and the ethics of care. Based on care ethics, the condition of humans as being needy and vulnerable also gives a basis for dealing with the political morality to care for all people living in society. In practice, undocumented migrants do not automatically have access to long-term health and social care. For example, this happens in Finland where undocumented migrants do not have public financial support for health care and have to pay the real costs. Finland received a notification from the European Committee of Social Rights (2013) for violating the right to the highest attainable standard of health for undocumented migrants.

With the second, care as a recognitive attitude requires concrete relations and the concentration to listen to the life situation in the person’s particular circumstances. Even if state policy does not really care what happens to human beings inside its territory, at least there should be public authorities, in co-operation with human rights’ organisations and other NGOs, who try their best to care for the needs of undocumented migrants and asylum seekers. The recognitive attitude of care incorporates both a cognitive and an emotional element (Ikäheimo, 2002). In the social work context, both elements are present. The cognitive element is there due to the science and ethical knowledge base of social work, which distinguishes social work from simply the goodwill of the person. Also, emotional elements cannot be disparaged in the mutual relationship of co-humans in social work encounters, where people are taken as persons and not just as instruments or people whose well-being does not matter.

With the third, in enabling care within the family of forced migrants, social workers must recognise the transnational nature of care relations. Migrants who have been settled for a long time outside their country of origin may entertain strong transnational social ties and practices (Faist *et al.*, 2013). Social workers must take into consideration the dual nature of transmigrants’ existence, while assessing and planning interventions and evaluating the services (Hunter *et al.*, 2010). Transmigrants do not only have acculturative stress in the host country but may also have enormous life stressors in the environment of their origin, such as war, family separation and family disintegration. One key problem in many countries is that the migrants’ possibilities for family reunification are prevented. This is the case in Finland, where family reunification prospects deteriorated after 2015 when more asylum seekers came than previously. According to a new act, a specific amount of income is required for the recognised asylum seeker if she or he wants to submit a family reunification application. For example, if a family consists of two adults and two children, the required income is 2,600 euros per month. The right to family life is a human rights’ issue. From the individual perspective, it is also a serious denial of relational care and love, and the denial of being considered as a person.

### Social esteem

Social esteem as a relation of recognition can be seen, first, as a right that concerns persons as individuals with their particularities, such as ethnicity and way of life. The right to maintain an ethnic identity that differs from the majority affects a person’s self-esteem (Ikäheimo, 2003). For Taylor and Gutmann (1994), esteem also takes a cultural dimension. With the demand for pluralism and individually chosen lifestyles, there is a need for positive value judgments from outsiders to maintain an individual or collective self-esteem (Seglow, 2009). Esteem recognition as a right for particular features could also be very relevant concerning a person’s own perceptions of identity formation and thus, self-realisation. For example, people from a refugee background may consider esteem as a right for their particularity, such as ethnicity, which is vital when they compare it to their past experiences. Ethnicity, religion or sexual orientation may be a reason for persecution and therefore, getting back such rights may be vital for person’s self-esteem. Accordingly, racism and xenophobic attitudes and acts show a serious disrespect in the sense of non-recognition.

Second, esteem can also be attained through personal accomplishments, such as goals, hopes, education and work, which need not be judged in a community (Seglow, 2009). The third dimension of esteem is closely related to personal accomplishments because it is a value we give to each other in terms of our speciality, such as capabilities or achievements. This is called the contributional dimension of esteem, which means valuing contributions to the common good without viewing another person as an instrument (Honneth, 1995). In social work, the identification of the potential and visions of forced migrants is vital, as this is what enables those visions to become reality (Niemi, 2015). Moreover, personal relations could be crucial in order to maintain hope or develop people’s strengths and opportunities in a new society. Often, recognition of past education and skills is needed before people can contribute to the common good of the particular society and also contribute in the global context, such as helping people in other societies using the transnational networks (Thompson, 2013). In addition, possibilities for contributions in transnational actions can help people’s own social recovery from past traumas and injustices (Turtiainen, 2012). In the actualisation of personhood, it is vital to have a possibility to orient oneself to the future. This possibility is denied for undocumented migrants and asylum seekers.

## Conclusion

The realisation of positive self-relations of asylum seekers and undocumented migrants is under a serious threat after they ask for refuge in European nation states. Nation states do not treat these people as persons deserving good life. Asylum seekers and undocumented migrants are often forced to survive in societies where their life, rights and contributions are not officially valuable to anyone. Besides, if they have family members in other countries, their possibility of maintaining family life could be very limited. Moreover, they are often victims of smugglers and are exploited by employers, to whom they are mere instruments and who disregard their human dignity. This is an everyday tragedy for millions of people worldwide.

Social work’s core obligation is to stay with these people and enable them to find ways of achieving full personhood and identity formation, which here becomes reality through the interpersonal relations of recognition. The recognition theory in social work practice for undocumented migrants and asylum seekers is best articulated by Ikäheimo’s (2003) understanding of rights concerning all the attributes of the person (needy being, autonomous and particular in a community). In social work practice, this means enabling rights for persons as human beings (enabling care relations), as autonomous (respecting rights) and having special features (social esteem through possible contributions to common good). In addition, there has to be a shift from nationally defined rights to the level of human rights (e.g. Thompson, 2013; Staub-Bernasconi, 2009, 2014; Ife, 2001). Respecting human rights instead of following national agendas could be the only possibility for undocumented people to gain self-realisation. This kind of understanding is vital in social work with undocumented people and asylum seekers.

In general, the concepts of the recognition theory are not new in social work practice but the theory collects all these relations together (Niemi, 2014). The recognition theory is a reminder of the different attributes of personhood (as a needy being, autonomous and particular in a community) and the normative criteria for communication (care, respect and esteem) in order to consider another person as a person like oneself, which, in turn, contributes to healthy self-relations. Furthermore, relations of recognition may contribute to social work by providing the sensitivity required to evaluate the complexity of explicit opinions, views and implicit background attitudes (Niemi, 2015), which are present as national agendas and which affect the way we encounter people. This paper shows that the context of these attitudes and values must be transferred from national to transnational settings and approaches in order to recognise asylum seekers and undocumented migrants.
